# SCO‐ph: Microfluidic Dynamic Phenotyping Platform for High‐Throughput Screening of Single Cell Acidification

**DOI:** 10.1002/smll.202504687

**Published:** 2025-06-16

**Authors:** Hyejoong Jeong, Emilia A. Leyes Porello, Jean G. Rosario, Da Kuang, Syung Hun Han, Jai‐Yoon Sul, Bomyi Lim, Daeyeon Lee, Junhyong Kim

**Affiliations:** ^1^ Department of Chemical and Biomolecular Engineering University of Pennsylvania Philadelphia PA 19104 USA; ^2^ Department of Biology University of Pennsylvania Philadelphia PA 19104 USA; ^3^ Department of Computer and Information Science University of Pennsylvania Philadelphia PA 19104 USA; ^4^ Department of Bioengineering University of Pennsylvania Philadelphia PA 19104 USA; ^5^ Department of Systems Pharmacology and Translational Therapeutics Perelman School of Medicine University of Pennsylvania Philadelphia PA 19104 USA

**Keywords:** extracellular acidification, glycolysis, high‐throughput, microfluidics, phenotyping, single cell

## Abstract

Studies on the dynamics of single cell phenotyping have been hampered by the lack of quantitative high‐throughput metabolism assays. Extracellular acidification, a prominent phenotype, yields significant insights into cellular metabolism, including tumorigenicity. Here, it is developed a versatile microfluidic system for single cell optical pH analysis (SCO‐pH), which compartmentalizes single cells in 140‐pL droplets and immobilizes ≈40,000 droplets in a 2D array for temporal extracellular pH analysis. SCO‐pH distinguishes cells undergoing hyperglycolysis induced by oligomycin A from untreated cells by monitoring their extracellular acidification. To facilitate pH sensing in each droplet, a cell‐impermeable pH probe is encapsulated and its fluorescence intensities are quantified. Using this approach, hyperglycolytic cells can be differentiated, and single‐cell heterogeneity in extracellular acidification dynamics can be concurrently observed. This high‐throughput system will be useful in applications that require dynamic phenotyping of single cells with significant heterogeneity.

## Graphical abstract

Single‐cell studies are on the rise; however, dynamic phenotyping studies remain challenging due to the lack of technologies. Single‐cell optical pH analysis (SCO‐pH) offers a high‐throughput, quantitative, and automated extracellular pH analysis platform based on microfluidic technologies. Temporal changes in extracellular pH are analyzed from ≈8,000 single cells at a time, providing a distinction of tumor‐like hyperglycolytic cells.

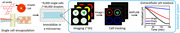

## 1 Introduction

Cellular acidification is a key indicator of mitochondrial respiration and glycolysis, giving an insight into cellular metabolic function. Typically, cancer cells are characterized by high glucose metabolism and lactate production through aerobic glycolysis, also known as the Warburg effect.^[^ ^1^ ^]^ Also, natural killer (NK) cells and T cells, the representative immune cells of adaptive cell transfer, are generally more glycolytic when proliferating and enhancing antitumor effects.^[^ ^2^, ^3^, ^4^ ^]^ Hence, the detection of cellular acidification is of great value in basic cell biology as well as for diagnosis, therapeutic monitoring, and efficient immune cell‐based cancer immunotherapy. Recently, the rise of single cell biology has shown that even for cells of the same genotype and same differentiated identity, there are individual variations leading to broad heterogeneity in many traits including metabolic functions such as acidification.^[^ ^5^, ^6^, ^7^, ^8^, ^9^, ^10^ ^]^ It is essential to quantify this heterogeneity because of its role in determining drug sensitivity, cellular response, phenotype, and anticancer efficacy of individual cells.

Fluorescence‐activated cell sorting (FACS) is a widely used tool for single cell phenotyping. FACS typically detects and sorts cells by their surface or intracellular fluorescent markers. However, this method is not suitable for secretion analysis, and is only a snapshot that does not contain dynamics over a period. Moreover, cells are fixed and no longer alive after FACS, limiting its utility. For dynamic analysis of cellular metabolism, Agilent Seahorse XF Analyzer is commonly used as a practical tool. It simultaneously measures extracellular acidification rates and oxygen consumption rates of live cells. Also, this instrument has the advantage of measuring cellular response in real‐time upon the addition of stimulants during cell culture and being a label‐free method. However, Seahorse is limited to bulk cell measurement and does not allow for single cell analyses.^[^ ^11^, ^12^, ^13^ ^]^

Droplet‐based microfluidic devices hold great promise as a powerful high‐throughput platform for single cell studies and biosensing because of their ability to allow compartmentalization of single cells into tiny chambers and analyses of metabolic products,^[^ ^14^ ^]^ proteins,^[^ ^15^, ^16^, ^17^ ^]^ nucleic acids,^[^ ^18^, ^19^, ^20^ ^]^ phospholipids^[^ ^21^ ^]^ and cellular mechanics.^[^ ^22^ ^]^ This technology has been successfully commercialized for single cell transcriptomics library for next generation sequencing.^[^ ^23^ ^]^ Single cell phenotyping can be achieved through observation of individual cells and their intracellular readouts. Moreover, droplet‐based methods allow isolation of individual cells in micro chambers, which in turn allows the detection of cells’ metabolic outputs into their surrounding media. For example, a method detecting circulating tumor cells by the measurement of single cell metabolism in droplet has been reported. As few as 10 cancer cells can be detected out of 200,000 white blood cells. Although high throughput and clinically applicable, this method requires a specially adapted microscope and does not provide droplet identification information.^[^ ^24^ ^]^ Another study has reported droplet‐based lactate quantification method from single cells by using a commercial lactate assay kit. However, this method relies on a complicated enzymatic reaction to detect lactate and only allows for measurements up to tens of minutes, thus limiting its utility in long‐term monitoring of cell metabolism.^[^ ^14^ ^]^

Herein, we develop a novel technology, SCO‐pH (single cell optical pH analysis microfluidic platform), based on our microfluidic technologies.^[^ ^25^, ^26^, ^27^, ^28^, ^29^, ^30^, ^31^, ^32^ ^]^ It is a high‐throughput single cell extracellular acidification analysis platform that co‐encapsulates single cells and a cell‐impermeant ratiometric pH probe, SNARF‐4F 5‐(and‐6)‐Carboxylic Acid. 40,000 droplets are positioned in a microwell array and monitored for at least 3 h in parallel. This technology offers several advances compared to previous studies: First, the microwell system assigns unique identifiers to droplets, facilitating their identification and isolation for subsequent analysis based on their acidification profiles. Second, this technique is straightforward to set up and implement from both physical and biological perspectives. A commercial pH probe that is robust and non‐toxic is used as a biological marker. Additionally, a standard inverted fluorescent microscope can be used with this technique, obviating the need for specialized modifications. Lastly, the automated data analysis pipeline is fully established, significantly reducing the analysis time for 40,000 droplets within a single day while enhancing data accuracy. Furthermore, the customizable scripts allow for modifications to minimize errors and adapt to various cell types, thereby increasing the versatility of the system. Building upon our prior success in selectively releasing droplets from a microwell array,^[^ ^33^ ^]^ we expand the functionality of the microwell array to enable pH quantification and automated data analysis, providing a powerful and versatile tool for cellular metabolic phenotyping and clinical cellular diagnosis.

## 2 Results and Discussion

### 2.1 Droplet‐Based Single Cell Extracellular pH Sensing

**Figure 1:**
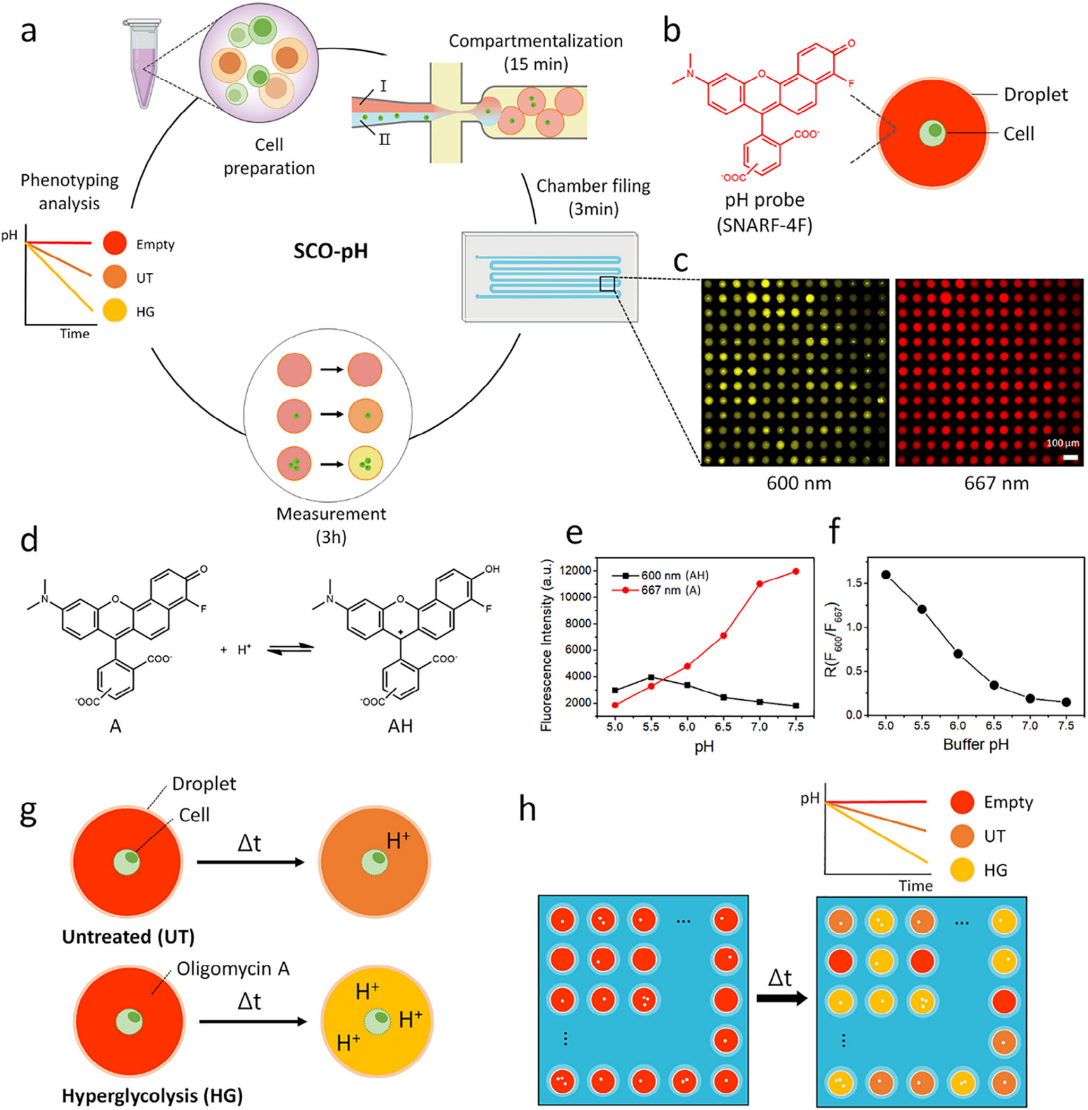
Principle and calibration of droplet‐based single cell optical pH analysis platform (SCO‐pH). a) Overview of the workflow and timing: EL4 cells are cultured and compartmentalized into 143‐pl droplets using a microfluidic device. Two aqueous phases (Phase I and Phase II) are utilized, with Phase I containing carboxy SNARF‐4F (a pH probe), as a fluorescently labeled detection reagent, and Phase II containing the cells. After generation, droplets are loaded into a microwell array for observation. Under static conditions without flow, extracellular pH and acidification rates are measured over time. A total of 40,000 droplets are observed through repeated imaging loops, displaying fluorescent intensities by incorporating carboxy SNARF‐4F and cells stained with dyes. The custom MATLAB code tracks and collects intensities of both droplets and cells. b) Droplet comprises single cell or multiple cells and a carboxy SNARF‐4F. c) Fluorescence images taken at 600 nm and 667 nm within a single imaging window, comprising 169 droplets among 240 positions in the array, show two emission intensities of carboxy SNARF‐4F. d) The acid (AH) and base (A) forms of carboxy SNARF‐4F at ground‐state equilibrium. e) Fluorescence intensities at 600 and 667 nm of 50 µm of carboxy SNARF‐4F at various pH values (ranging from 5 to 7.5). The “AH” form exhibits an emission peak at 600 nm, while the “A” form emits at 667 nm. f) The ratio of fluorescence intensities at 600 and 667 nm (R(F_600_/F_667_)) at various buffer pH. g) Schematic illustrations depicting changes occurring in droplets containing untreated (UT) and hyperglycolytic (HG) single cells. h) Representation of a microwell array with droplets displaying varying acidification levels.

The general principle and workflow of SCO‐pH is schematically illustrated in **Figure** **1**a. Single cells are compartmentalized in monodisperse aqueous droplets with 60 ≈ 65 µm diameter (113 ≈ 143 pL in volume) in an inert fluorinated oil using a microfluidic flow‐focusing device (Figure 1a; Figure S1, Supporting Information). Reagents and cells are injected into the microfluidic flow‐focusing device, generating droplets at a rate of ≈1,000 water‐in‐oil single emulsions per second. The cell concentration adjusted to generate up to 30% of droplets containing single cells (300 single cell droplets/sec), following the Poisson distribution; 18% and 52% of droplets are empty and contain multiple cells, respectively. The droplets are uniform, with an average diameter of 59.9± 2.4 µm and a 4.0% of polydispersity (Figure S2, Supporting Information). The generated droplets subsequently flow into a device with a serpentine channel, where the top surface features a 2D microwell array. As they flow through the channel, droplets float into the microwells due to the density difference between water (ρ = 1.0) and oil (ρ = 1.614) (Figure S3, Supporting Information). ≈40,000 droplets (i.e., ≈12,000 single cell droplets) are captured in the microwell array within 3 min. A 10× objective lens can capture 13 × 13 microwells in a single imaging window; each well in each image is indexed for kinetic analysis of acidification. An automated microscope stage is controlled to image the entire microwell array device across 240 positions, capturing images of all 40,000 microwells within 15 min.

To measure the extracellular pH within each droplet, we co‐encapsulated cells with a cell‐impermeable pH probe, carboxy SNARF‐4F, in the droplets (Figure 1b). Carboxy SNARF‐4F exists in acid (AH) and base (A) forms, with emission peaks at 600 and 667 nm, respectively (Figure 1d). Carboxy SNARF‐4F has different fluorescence intensities at these two wavelengths depending on the pH condition (Figure 1e). The ratio of fluorescence intensities at 600 and 667 nm is a linear function of the pH of the solution between pH 5.0 ≈ 6.5 (Figure 1f). Ratiometric fluorescent probes offer built‐in self‐calibration for signal correction by measuring the ratio of intensity profiles across different wavelengths. This process enables a more reliable determination of pH.^[^ ^34^ ^]^ The spectral shift means that measurements at the two wavelengths can be used to compensate for sample‐to‐sample variations caused by dye concentration or path length. This ratiometric method helps control multiple artifacts in fluorescence measurements, including instrument stability and non‐uniform indicator loading. To improve the accuracy of the data, we use 555 nm laser for excitation, as this wavelength corresponds to the maximum absorption of the pH probe at different pH values (Figure S4, Supporting Information). In addition, we set up band‐pass filters centered at 600 and 667 nm to measure the intensities of two emissions of carboxy SNARF‐4F, as shown in Figure 1c. Additional information on the filter sets and carboxy SNARF‐4F is provided in the Supplementary information (Figure S5, Supporting Information). We use a stage‐top environmental chamber to maintain the cell culture environment at 95% humidity and 37 °C. The configuration of the inverted microscope covered with the environmental chamber is shown in Figure S6 (Supporting Information).

To test extracellular acidification monitoring at the single‐cell level, we developed a hyperglycolytic (HG) cell, which exhibits overactivated glycolysis upon treatment with oligomycin A in the droplets (Figure 1g). The droplets containing untreated cells and those containing the HG cells are mixed and captured in the microwell array. The fluorescence intensity ratio of F_667_ and F_600_ from each droplet changes depending on extracellular pH, which is influenced by glycolysis and mitochondria‐derived acidification of the two cell types (Figure 1h).

### 2.2 Buffer and CO_2_ Condition for Extracellular pH Measurement

To accurately measure the extracellular pH of each droplet, it is critical to ensure that environmental factors do not affect the medium pH and at the same time the pH probe respond sensitively to the pH change caused by cell metabolism. The pH of culture media in conventional cell culture experiments is maintained around 7.4 by the equilibrium between NaHCO_3_ in the liquid media and 5–6% CO_2_ in the gas phase.^[^ ^35^ ^]^ We, however, find that this conventional approach is problematic because the pH of droplets devoid of cells decreases rapidly when the CO_2_ concentration is maintained at 5% in the buffered solution (Figure S7, Supporting Information). To address this issue, we used a medium condition optimized for the Agilent Seahorse (Santa Clara, CA). This system employs 0% CO_2_ condition during extracellular pH measurement, along with their buffer system (Seahorse XF medium), which is free of bicarbonate and contains no phenol red. Because phenol red absorbs wavelengths of 450 and 560 nm, it overlaps with the pH probe and might affect the sensitivity of pH measurement. We maintain the CO_2_ concentration within the environmental chamber at 0%, which is continuously tracked using the monitoring system (Figure S6c, Supporting Information). According to the supplier, this buffer solution can be used for up to 6 h and eliminates potential problems that may impact the cellular metabolism.

To determine the suitability of using this Seahorse buffer system for measuring a subtle change in the pH in these pico‐scale droplets, we compared phosphate buffered saline (PBS), live cell imaging solution (LCIS), and the Seahorse XF RPMI medium for droplet‐based extracellular pH measurement. The compositions of three different buffers are provided in the Supplementary information (Table S1, Supporting Information). To test these three buffers, we measured fluorescence intensities of carboxy SNARF‐4F in four different conditions, including 0.1X PBS, XF medium, XF medium with supplements (containing GlutaMax, glucose, and pyruvate following the pH measurement condition of Seahorse technology),^[^ ^36^, ^37^ ^]^ and LCIS (0% CO_2_, 37 °C, 95% humidity). We find that the fluorescence intensities remain most stable in the supplemented XF medium compared to other buffers (Figure S8, Supporting Information).

### 2.3 Droplet Crosstalk and Stability

**Figure 2:**
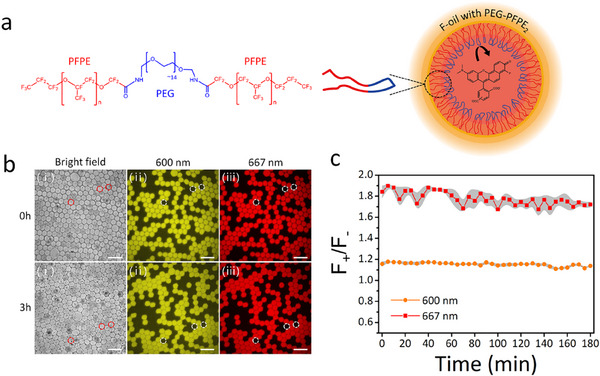
Droplet crosstalk and stability test. a) Chemical structure of EA‐surfactant, and schematic illustration of EA‐stabilized droplet in the fluorinated oil phase containing carboxy SNARF‐4F. b) Fluorescence images of “positive” and “negative” droplets stabilized by EA‐surfactant after mixing (0 h) and after 3 h. The emulsion contained a 1:1 mixture of “positive” droplets containing carboxy SNARF‐4F (50 µm) and “negative” droplets containing buffer only. i) Bright‐field image, fluorescence images at ii) 600 nm and iii) 667 nm. Three negative droplets are marked with red and white dashed circles. Scale bar is 200 µm. c) Time evolution plots of the ratio of fluorescence intensity of positive droplets (*F*_+_) versus that of negative droplets (*F*_‐_) at 600 and 667 nm. The mean value is calculated from random three droplets in each image and the grey area represents standard deviation of the mean (*n* = 3).

To ensure that cell metabolism in a particular droplet affects only the pH of that droplet and not any others, it is crucial that the water droplets retain carboxy SNARF‐4F without compromising emulsion stability and that the probe does not transfer between droplets under the experimental conditions of 37 °C, 95% humidity, and 0% CO_2_. EA‐surfactant is a non‐cytotoxic triblock copolymer composed of poly (ethylene glycol) and perfluoropolyether (PEG‐PFPE_2_) and is widely used to stabilize aqueous droplets to encapsulate cells (**Figure** **2**a). However, poor quality control during manufacturing^[^ ^38^, ^39^ ^]^ can compromise the stability of EA‐surfactant stabilized emulsions; moreover, small molecules (≈200 to ∼500 Dalton) can pass between EA‐stabilized droplets due to the presence of micelles in the oil phase.^[^ ^40^, ^41^, ^42^ ^]^

To test whether these issues could complicate our approach, we performed experiments to test the stability of emulsion droplets and the possible presence of probe transfer between droplets. We produced and mixed two types of droplets: “Positive” droplets containing carboxy SNARF‐4F and “negative” droplets with buffer only. These two types of droplets are mixed a 1:1 volume ratio and observed in a PDMS‐based chamber for 3 h (Figure 2b). We decided to observe the cellular acidification changes for 3 h, referencing Agilent Seahorse technology, which typically measures cellular metabolism for 2 to 3 h. Three negative droplets are marked with a red dashed circle in the bright field image and white dashed circles in the fluorescent images at 600 nm and 667 nm. Negative droplets remain empty and stable with no size, shape, or color changes, indicating no crosstalk among the droplets stabilized with EA surfactant. The leakage of carboxy SNARF‐4F is characterized by measuring the fluorescence intensity ratio (*F*_+_/*F*_‐_) between carboxy SNARF‐4F in positive and negative droplets over time (Figure 2c). The ratio (*F*_+_/*F*_‐_) indicates that two different forms of carboxy SNARF‐4F remain stable in droplets stabilized by EA‐surfactant in HFE‐7500 for 3 h.

### 2.4 Cell Model with Over‐Activated Glycolysis

**Figure 3:**
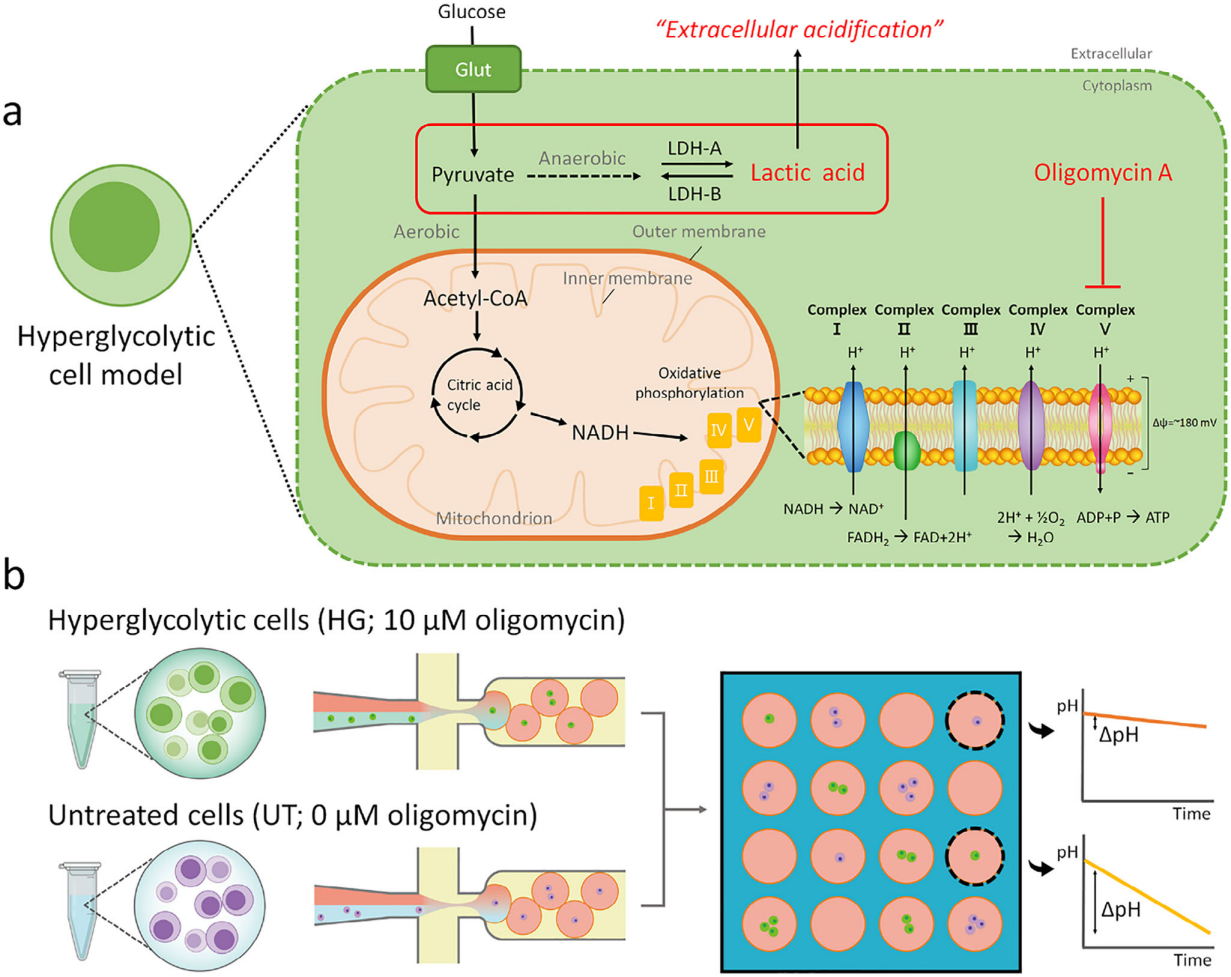
Oligomycin A‐induced hyperglycolytic cell model and experimental design. a) The hyperglycolytic (HG) cell model induced by oligomycin A, and cellular mechanism of extracellular acidification. b) Experimental design for distinguishing HG cells from untreated (UT) cells in a microwell array. The HG cells exhibit green fluorescence, while the UT cells display violet fluorescence. Each cell type is compartmentalized separately into droplets, which are then mixed and randomly loaded into the microwell array for observation.

To determine that SCO‐pH accurately monitors the extracellular acidification of single cells, we over‐activated glycolysis in cells by using oligomycin A. This is an inhibitor of ATP synthase and significantly suppresses mitochondrial respiration and maximizes glycolysis by blocking the proton channel of ATP synthase.^[^ ^43^ ^]^ We expect that oligomycin A‐treated cells would exhibit high levels of lactate production, resulting in rapid extracellular acidification of the aqueous droplets (**Figure** **3**a). This hyperglycolytic (HG) cell model is compared to the control group (UT; oligomycin A untreated group). Although ionophores could theoretically equilibrate intra‐ and extracellular pH, they were not applied in our system because they could make cells more fragile, potentially affecting cellular metabolism. In addition, since nearly all cells have transmembrane voltages in the range of ‐60 to ‐90 mV, they accumulate H^+^ at levels 10 to 30 times higher than the extracellular pH.^[^ ^44^ ^]^ Moreover, the ionophore A23187 promotes the uptake of lactate rather than its release by increasing intracellular Ca^2+^ concentration.^[^ ^45^ ^]^ Therefore, we expect that cells treated with ionophores would exhibit different acidification patterns and disrupt the measurements of inherent cellular metabolism.

HG cells are prepared by staining EL4 cells with Calcein AM and co‐encapsulating them with 10 µm of oligomycin A and 50 µM of carboxy SNARF‐4F as shown in Figure 3b. To distinguish the droplets containing UT cells from those containing HG cells, EL4 cells are stained with CellTrace violet and encapsulated without oligomycin A. Two different droplets are mixed in a 1:1 volume ratio and randomly immobilized into the microwell array. With this approach, we can collect the acidification dynamics of each cell using two different sets of images, tracking only green‐labeled cells or violet‐labeled cells.

### 2.5 pH Measurement by Droplet Image Analysis

**Figure 4:**
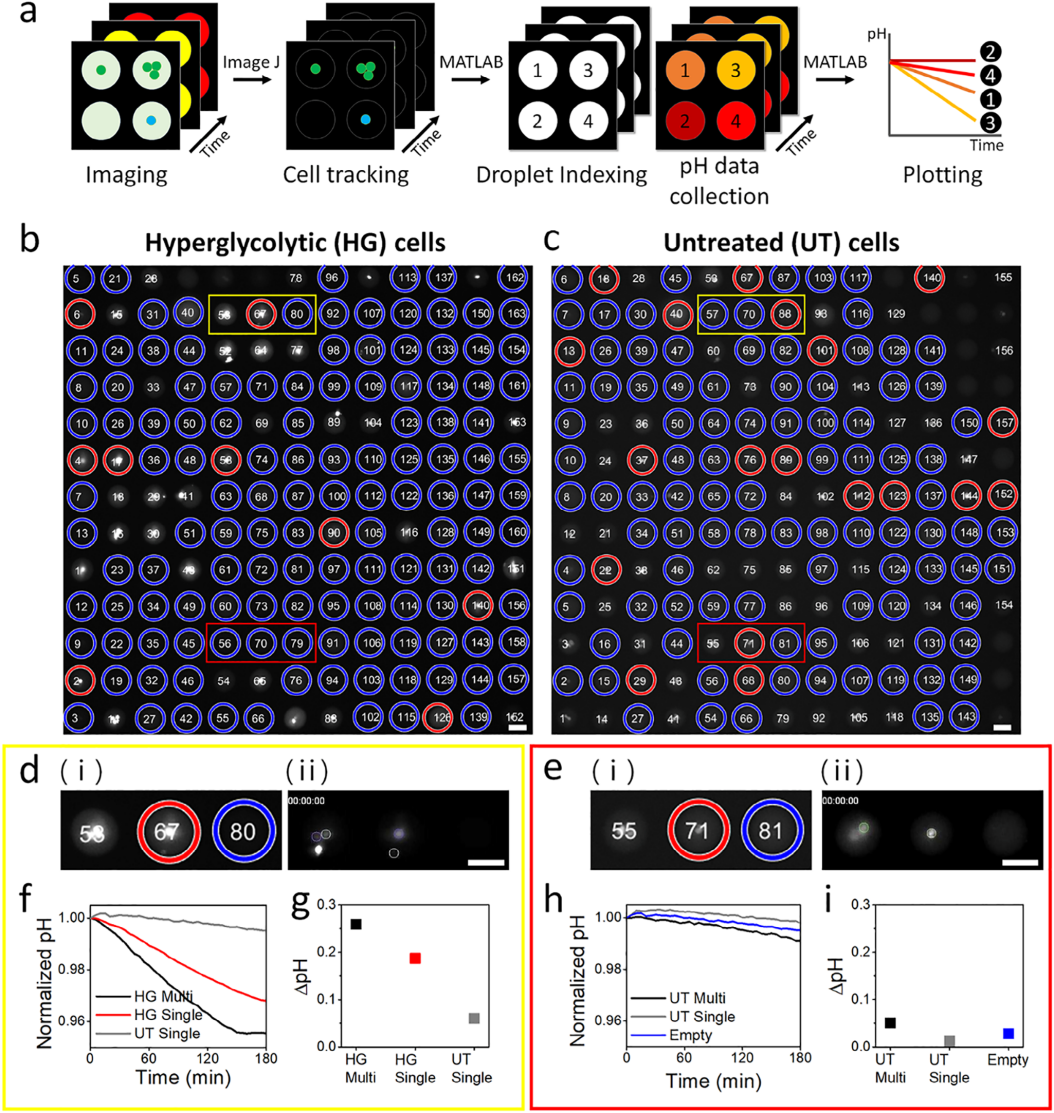
Droplet indexing and extracellular pH collection. a) Workflow of image analysis using the “Particle Tracker” of FIJI for cell tracking, followed by droplet indexing, extracellular pH collection, and plotting using custom MATLAB codes. Droplet indexing results obtained from MATLAB: b) Hyperglycolytic (HG) cells are detected in the green channel, and c) untreated (UT) cells are detected in the UV channel. Droplets containing a single cell are marked with a red circle, empty droplets are marked with a blue circle, and droplets containing multiple cells are unmarked. For examples of pH monitoring performance, three droplets within the yellow (Position 1) and red (Position 2) boxes in both images are magnified in d) and e), respectively. (d) i) Magnified images of droplet indexing for HG cells and UT cells, along with ii) images of detected cells by the Particle Tracker, are displayed. Single cells detected in the representative image at the start of imaging (0 h) are outlined. e) i) Magnified images of droplet indexing for HG cells and UT cells are shown in red boxes. ii) The image of detected cells obtained by the Particle Tracker is displayed. Cells are outlined in the image captured at the start of imaging (0 h). Color coding for f) to i): HG single cell droplet (red), HG/UT multiple cell droplet (black), UT single cell droplet (grey), and empty droplet (blue). (f) Normalized pH and g) raw ΔpH (initial pH‐final pH; pH_i_‐pH_f_) for the droplets shown in (d; Position 1) over 3 h. h) Normalized pH and i) raw ΔpH (initial pH‐final pH; pH_i_‐pH_f_) for the droplets shown in (e; Position 2) over 3 h. The scale bar is 50 µm. Each data point was obtained from a single measurement.

Images of the captured droplets in the microwell array are automatically taken by the motorized microscope stage operated by a custom script of Metamorph software (Molecular Devices, San Jose, CA). The image analysis pipeline is shown in **Figure** **4**a. Cell tracking in the droplets is performed by the free software FIJI with a Particle Tracker customized for automatic image analysis. To monitor the extracellular pH of individual droplets, we employ custom MATLAB scripts. These scripts facilitate droplet indexing and enable the discrimination single cell droplets, multiple cell droplets, and empty droplets based on cell tracking data using a custom MATLAB script. The results of this image analysis enable the identification of relevant droplets within the image and the tracking of their pH variations. Our MATLAB script can be tailored to generate graphical representations, including plots depicting droplet intensities, cell intensities, and extracellular pH values.

The droplet indexing protocol identifies droplets containing single cells with red outlines and empty droplets with blue outlines. Droplets with no outlines represent multiple cell droplets. Droplet indexing outputs in a representative position confirm that the image pipeline reliably works for both images regardless of which dye the cells are stained with (Figure 4b,c). As an example, we observe three droplets in a yellow box that has been placed in an arbitrary location for both HG and UT cells (Figure 4d). The image of outlined cells by the Particle Tracker (Figure 4d, (ii)) is displayed next to the droplet indexing image (Figure 4d, (i)). In the droplet containing single cell (middle), the droplet boundary is detected as a cell. We find that the Particle Tracker is prone to error near the outer boundaries of each image as it misidentifies high contrast patches at the edge of droplets as cells. Therefore, we analyze the central 60% of the droplet area to identify the number of cells in each droplet. Upon applying the code to the six droplets in the yellow boxes (3 HG and 3 UT droplets), we successfully identified one HG multiple cell, one HG single cell, and one UT single cell (Figure 4d; Figure S9, Supporting Information). As shown here, we are able to detect that the empty HG droplet is not actually empty and is a UT single cell droplet by comparing the two images. In another location marked using red boxes, no HG cells are found, and only UT cells are detected. The first droplet contains UT multiple cells, and the second droplet contains a UT single cell. The third droplet is empty as it appears empty in both images (Figure 4e). Additionally, we provide cell tracking videos of those droplets in the Supplementary Information (Video S1, Supporting Information).

To verify the validity of SCO‐pH for extracellular pH monitoring of single cells, we characterize the pH curves from each droplet based on the MATLAB outputs. The first location (yellow box) shows different acidification dynamics among the droplets. A HG single cell droplet shows drastic extracellular pH reduction over time compared to a UT single cell droplet. Also, the HG multiple cell droplet shows faster acidification than the HG single cell droplets, as expected (Figure 4f). The actual pH of the droplets can be calculated based on a calibration curve created by an equation provided by the manufacturer of carboxy SNARF‐4F (Figure S10, Supporting Information). The normalization factor—a constant derived from the fluorescence intensity ratio at 667 nm at the acidic and basic endpoints—is accounted for in the pH calculation. This factor enhances the accuracy of the pH measurements. To verify how much acidification occurs in the droplet by cells, we compare raw ΔpH (initial pH‐final pH) of each droplet based on the pH calculation (Figure 4g). Raw ΔpH of each droplet containing the HG multiple cells and the HG single cell decreases by 0.26 and 0.19, respectively. In contrast, the UT single cell droplet shows only 0.06 raw pH shift in 3 h. Another example in a different location (red box) clearly shows that in the case of the UT cell droplets, extracellular acidification is not different between the multiple cell droplet and the single cell droplet. Normalized pH plots show that all plots have similar trends among the UT single cell droplet, the UT multiple cell droplet, and the empty droplets (Figure 4h). Extracellular raw pH decreases by only 0.05, 0.01, and 0.03, respectively (Figure 4i). While the discrepancy between the HG single‐cell droplet and the HG multiple cell droplets may appear small, significant differences in acidification across the three positions and the entire microarray (240 positions) are observed as discussed in the later sections. It is essential to demonstrate the effectiveness of the SCO‐pH technology in distinguishing acidification within single‐cell droplets from that occurring in multiple cell droplets.

### 2.6 Correlation Analysis Between Extracellular pH and Glycolysis

Our results above demonstrate that SCO‐pH detects extracellular pH in droplets reliably at single cell levels. Additionally, our custom image analysis pipeline rapidly and accurately tracks the pH of droplets and classifies droplets. Using SCO‐pH, we analyzed all the droplets in three positions out of 240 positions in the entire 40,000 microwell array to validate the acidification monitoring performance at the single cell level. A total of 507 droplets were analyzed from the three positions. In these areas, single cell encapsulation rate is 17.6%, less than the expected calculation (30%). We observed two types of cells with green and violet fluorescence, and some highly fluorescent cells generate outlier errors in the data. After eliminating errors, the single cell encapsulation rate that can be identified through our code is 11.8%.

**Figure 5:**
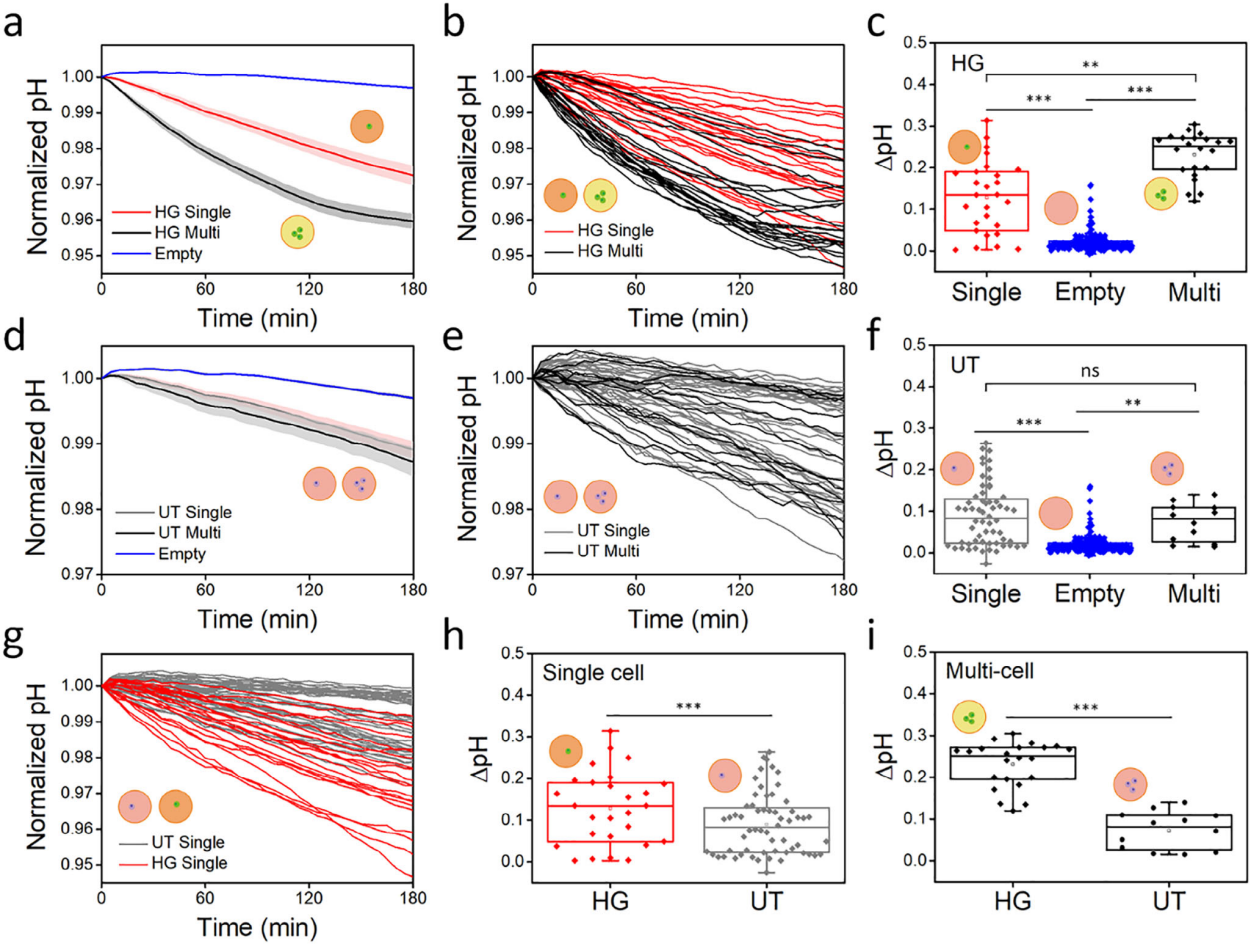
Extracellular pH differences in cell models with different acidification observed for 3 h in three different positions in a device. The hyperglycolytic (HG) cell model analysis: a) Normalized and averaged extracellular pH of the droplets containing single cells (red, n = 21), multi‐cells (black, n = 22), and no cells (empty; blue, n = 219) tracked for 3 h. Shaded regions correspond to standard error. b) Individual normalized extracellular pH curves of the droplets with single cells and multi cells. c) Raw ΔpH (initial pH‐final pH; pH_i_‐pH_f_) of the droplets containing single cells, multi‐cells, and no cells for 3 h. The untreated (UT) cell analysis: d) Normalized and averaged extracellular pH of the droplets containing single cells (*n* = 39), multi‐cells (*n* = 12), and no cells (*n* = 216) tracked for 3 h. Shaded regions correspond to standard error. e) Individual normalized extracellular pH curves of the droplets containing single cells and multi‐cells. f) Raw ΔpH of the droplets containing single cells, multi‐cells, and no cells for 3 h. Comparison of the HG single cell droplets (red) and the UT single cell droplets (grey): g) Normalized extracellular pH curves of the individual droplets containing the HG and the UT single cells. h) Raw ΔpH of the droplets containing the HG and the UT single cells. i) Raw ΔpH of the droplets containing the HG and the UT multi‐cells. The significance of the data is calculated by the two‐tailed Student's *t*‐test (ns, *p* > 0.05; ^*^, *p*  ≤ 0.05; ^**^, *p* ≤ 0.01; ^***^, *p* ≤ 0.001).

We compare the extracellular acidification of single and multiple cells in the HG and the UT cell models. The normalized pH plots of droplets containing HG cells show different acidification patterns among single cell droplets, multiple cell droplets, and empty droplets (**Figure** **5**a). Also, the pH curve distribution of individual droplets indicates compartmentalized patterns (Figure 5b). Multiple cells decrease the pH of the droplet more rapidly compared to single cells. In comparison of ΔpH, the extracellular pH of single cell droplets decreases by 0.17 ± 0.07 units over a span of 3 h. This value is 8 times higher than that for empty droplet and 0.7 times lower than that for multiple cell droplets. ΔpH of single cell droplets is also significantly different from multiple cell droplets and empty droplets (Figure 5c).

In the case of the UT cells, single cell droplets and multiple cell droplets are not significantly different in the averaged pH curves and individual pH curve distribution (Figure 5d,e). Nonetheless, single cell droplets and multiple cell droplets differ significantly from empty droplets due to glycolysis (Figure 5f). Given the absence of disparity in acidification between single cells and multiple cells, the impact of acidification on UT cells appears to be minimal.

Finally, we compare the ΔpH of the droplets containing single cells to assess the utility of this technology in analyzing the heterogeneity of individual cells. The pH distribution of single cell droplets reveals different patterns for HG and the UT cells (Figure 5g). Generally, the red plots representing HG single cell droplets exhibit a more rapid decreasing trend compared to the grey plots of UT single cell droplets. Nevertheless, individual cells have different acidification dynamics with fair variation in the rates of acidification, including some HG cells showing slower decrease than control cells, representing single cell heterogeneity in metabolism. These results indicate the capability of SCO‐pH to measure extracellular acidification at single cell level and assess population heterogeneity. Overall, extracellular pH change of HG single cell is 0.17 in 3 h, which is 2.6 times higher than the one for the UT cells (Figure 5h). In the case of multiple cell droplets, the extracellular pH change of the HG is 0.23 in 3 h, 3.2 times higher than that of the UT cells (Figure 5i).

### 2.7 Extracellular pH Monitoring Performance of the Entire Device

**Figure 6:**
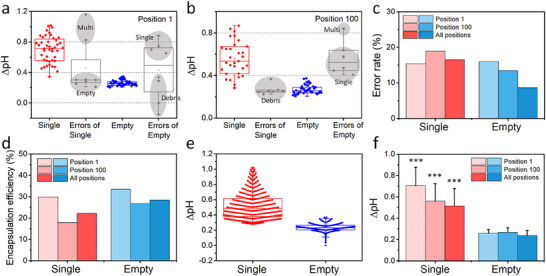
Extracellular pH monitoring performance of an entire device (239 positions): error rate, encapsulation efficiency, and extracellular pH change of single cells for 3 h of glucose consumption. a) Representative raw ΔpH and error distribution of single cell droplets and empty droplets in Position 1. b) Representative raw ΔpH and error distribution of single cell droplets and empty droplets in Position 100. c) The error rates for single cell droplets and empty droplets in Position 1, 100, and all 239 positions. d) Encapsulation efficiency of single cell droplets and empty droplets in Position 1, 100, and all 239 positions. e) Raw ΔpH of single cell droplets (*n* = 8,600 of 38,759 droplets analyzed) and empty droplets (*n* = 11,012 of 38,759 droplets analyzed) across all positions. f) Raw ΔpH values of single cell droplets and empty droplets measured at Position 1, Position 100, and across all 239 positions. Sample sizes are as follows: Position 1 (*n* = 44/50), Position 100 (*n* = 30/45), and all positions combined (*n* = 8,600/11,012) for single cell droplet/empty droplet, respectively. The significance of the data is calculated by the two‐tailed Student's *t*‐test (^***^, *p* ≤ 0.001).

Finally, we analyze the extracellular pH dynamics of EL4 cells in the glucose‐rich environment (10 mm) across all positions in the microwell array (239 positions). To quantify the error rates of this technology, we manually find errors (i.e., those identified as “single cells” containing multiple cells or no cell, or those identified as “empty” containing one or more cells) in Position 1 and 100. The error rates in Position 1 are 15.4% and 16.0% for single cell droplets and empty droplets, respectively. In the case of single cell droplets, high values of raw ΔpH among outliers over 0.83 are identified as multiple cell droplets, and low values under 0.28 are empty droplets containing debris. For empty droplets, low values among outliers are identified as debris that is not a cell and droplets stuck in the wrong area. High values are from single cell droplets misidentified due to cells having low fluorescence intensity (**Figure** **6**a). We analyze Position 100 as another representative example to quantify the error rates in the same way. The error rates are 18.9% and 13.5% for single cell droplets and empty droplets, respectively. We find that if the ΔpH of empty droplets are greater than 0.41, it is due to single cell droplets and multiple‐cell droplets containing cells with weak fluorescence intensities. For single cell droplets, low ΔpH under 0.37 is identified as debris, not cells (Figure 6b).

According to the manually analyzed results, we regard the data in the range lower than 0.37 as empty droplets and the data in the range of 0.28–1.02 as single cell droplets. After applying this criterion, we obtain 11,012 empty droplets and 8,600 single cell droplets out of 38,759 droplets in the microwell array. The error and encapsulation rates of single cell droplets are 16.5% and 22.2%, respectively. It is estimated that the debris of empty droplets causes 88.6% of the errors (ΔpH < 0.28). Only 11.4% of errors are estimated to be misidentified multi‐cell droplets (ΔpH > 1.02). The error and generation rates of the empty droplet are 8.7% and 28.4%, respectively. We estimate that most errors are droplets containing cells with low fluorescence intensity (ΔpH > 0.37). The error and encapsulation/generation rates are similar to the values for Position 1 and 100 (Figure 6c,d). For the single cell droplet, encapsulation efficiency reaches 22.2% after eliminating the errors, and we still obtain data for over 8,000 single cells. Although the error rate is 16.5%, the remaining data is close to what we aimed for based on the calculation (30%, 12,000 single cells) and will be sufficient to determine the metabolic characteristics of single cells.

Raw ΔpH distributions of single cell droplets and empty droplets in all positions are displayed, and the two data exhibit different median values and distribution patterns (Figure 6e). Furthermore, we can see the metabolic heterogeneity of single cells through a large distribution area. The average raw ΔpH values of single cell droplets and empty droplets across all positions are similar to those at Position 1 and 100, and exhibit a significant difference due to the cellular acidification (Figure 6f). Taken together, we demonstrate that SCO‐pH is able to perform various tasks from imaging to analysis successfully throughout the entire microwell array to track extracellular pH over time, and thus be useful for applications that require dynamic monitoring of acidification at the single cell level.

## 3 Conclusion

As a single‐cell phenotyping tool, SCO‐pH effectively measures the temporal acidification dynamics of individual cells in thousands of droplets over a 3‐h period. To ensure the robustness of this technique, we have carefully validated droplet stability, buffer conditions, and the stability of the pH probe. Cells are compartmentalized into picoliter‐scale droplets, which are captured in a 2D array to provide temporal readouts. Within each imaging window, the pH of the droplets, equivalent to the extracellular pH, is accurately tracked using the intensity ratio between two emissions of the pH probe. Subsequently, droplets are assigned unique identifiers, and their pH profiles are categorized into empty, single cell, and multiple cell groups based on the cell count within each droplet. The entire data analysis process is automated using a series of custom scripts. This approach enables the identification of droplets with anomalous acidification profiles within the image or microwell device. By excluding outlier data and refining the data analysis pipeline, we significantly enhance data accuracy. SCO‐pH successfully differentiates the acidification patterns of HG and UT cells, while also elucidating their single‐cell heterogeneity.

We believe SCO‐pH represents a significant advancement over previous methodologies in tracking the extracellular pH of single cells and observing the temporal dynamics of pH changes due to its simplicity, capacity, and low cost. This technique holds promise in the development of cancer immunotherapy, especially adoptive cell therapy. During in vitro expansion procedures, NK cells and T cells require approximately three weeks and several days, respectively. Metabolism has emerged as a crucial determinant of NK/T cell fate and function, essential for improving the antitumor efficacy of these cells. The Seahorse XF technique, which measures cellular metabolism at intervals of several days during long‐term expansion, is limited by the requirement for expensive kits and specialized equipment. In contrast, SCO‐pH requires only a standard fluorescent microscope, rendering it more cost‐effective and suitable for routine application. More significantly, our technique allows for the analysis of individual cells, a capability not feasible with Seahorse XF. This capability could facilitate the assessment of cellular quality and phenotype as therapeutic agents. For clinical applications, analyzing the metabolic characteristics of NK/T cells could allow for monitoring a patient's immune response to anticancer therapy, facilitating more personalized treatment. We note that such applications will require further calibration of external conditions including the optimization of the buffer.

We note that while we developed the current application using an extra‐cellular pH reporter, the overall platform consisting of microfluidics‐based large‐scale optical static array can be applied to many other kinds of metabolic reporters with optical readout. In addition, our system could be extended to physically collect selected droplets that exhibit unique trends using a previously developed laser‐based method.^[^ ^33^ ^]^ Integrating a sorting step could further enhance the SCO‐pH devices’ ability to monitor a larger number of individual cells, thereby improving statistical robustness. Moreover, the microwell array can be easily scaled up to accommodate the observation of a substantial number of cells—potentially beyond 40,000—concurrently, thus facilitating the screening of rare cells, such as circulating tumor cells, for enhanced cancer diagnosis. The robust performance, economic efficiency, and ease of use of SCO‐pH strongly suggest that it can be commercialized as a first‐line tool for single‐cell phenotypic measurements.

## 4 Experimental Section

### Reagent

Fluorinert FC‐40, trichloro(1H,1H,2H,2H‐perfluorooctyl)‐silane (PFOTS), and oligomycin A were purchased from Sigma–Aldrich (MO, USA). HFE‐7500 was obtained from 3 m (MN, USA). FluoSurf 2wt.% in HFE‐7500 was obtained from Dolomite Microfluidics (Royston, UK). Sylgard 184 silicone elastomer kit was purchased from Dow Corning (MI, USA). Invitrogen SNARF‐4F 5‐(and‐6)‐Carboxylic Acid, CellTrace Violet, Calcein AM, and live cell imaging solution were purchased from Invitrogen (MA, USA). Seahorse XF RPMI assay medium pack is purchased from Agilent Technologies (CA, USA).

### Device Fabrication

Devices for droplet generation (called “droplet generator”) and trapping droplets (called “microwell array”) were fabricated using a previously reported method.^[^ ^33^ ^]^ The device masks were designed by AutoCAD 2018 and printed on Cr masks by CAD/Art Service, Inc. (CA, USA). A master mold was fabricated on a 3″ silicon wafer (University Wafer Inc., MA, USA) using the conventional soft lithography technique. The master molds were all fabricated inside a cleanroom in the Quattrone Nanofabrication Center of the Singh Center of Nanotechnology at the University of Pennsylvania. The droplet generator was fabricated via the single‐layer soft lithography technique. A negative photoresist SU‐2025 (MicroChem, MA, USA) and a film mask were used, and the thickness of the droplet generator mold was 40 µm controlled by adjusting the rotation speed of spin coating in conjunction with the UV exposure time under a mask aligner (ABM 3000HR Mask Aligner, ABM, NY, USA). To produce the master for the bottom trap channel of a microwell array, a multilayer mold fabrication method was employed using Cr photomasks prepared by a laser writer (Heidelberg DWL 66+ Laser Writer, Heidelberg Instruments, Germany). Multilayer mold fabrication skipped mold development after initial post‐bake and proceeded with a spin coating of the second photoresist layer. The top flow channel mold was fabricated via the single‐layer soft lithography technique. Fabricated master molds were subsequently silanized with PFOTS to facilitate the detachment of cured polydimethylsiloxane (PDMS). PDMS precursor was prepared by mixing the base and curing agents of Sylgard 184 in a 10:1 ratio and was degassed in a vacuum chamber for 30 min.

The degassed PDMS mixture was poured onto the master mold. The thickness of poured PDMS for a droplet generator and a microwell array was ≈3 mm. After an additional degassing step for 30 min, PDMS was cured in an oven for 4 h at 65 °C. The PDMS molds were bonded to a plain glass slide using a conventional oxygen plasma treatment.

### Droplet Generation and Trapping

The droplet generator made with PDMS was silanized with a 2% PFOTS solution in HFE‐7500 oil for 5 min following plasma treatment. The device was flushed with neat HFE‐7500 oil, then connected with three polytetrafluoroethylene (PTFE) tubing lines, each linked to a syringe. One syringe, filled with HFE‐7500 containing 2 wt.% EA‐surfactant, was connected to an oil inlet at the top of the generator. The other two syringes, filled with an aqueous solution containing cells or carboxy SNARF‐4F dye, were connected to water inlets in the middle of the device. The EA surfactant is a commercially available tri‐block copolymer composed of perfluoropolyether (PFPE) and polyethylene glycol (PEG) blocks and is most widely used in emulsion stabilization in fluorinated oils. By connecting syringes to two syringe pumps (Harvard Apparatus, MA, USA) for each oil and aqueous solution, flow rates of 150 and 300 µL h^−1^ were used for the oil and aqueous phases, respectively. In this condition, uniform droplets with ≈60 ∼ 65 µm in diameter were generated. Prior to droplet introduction, a microwell array was silanized with a method the same as above and flushed with neat HFE‐7500 oil for 2 min to remove air bubbles within the device. Droplets generated from the droplet generator traveled through a PTFE tubing and immediately entered a microwell array device. With >60% of the wells filled with droplets, the droplet injection tubing was disconnected, and neat FC‐40 was injected slowly (200 µL h^−1^) to fill the remaining wells with the droplets. Afterward, neat FC‐40 oil was injected rapidly (500 µL h^−1^) to remove untrapped droplets from the channel.

### Droplet Crosstalk Test

Two different ensembles of droplets were generated using droplet generators: one filled with only Seahorse XF RPMI medium supplemented with 1 mm of pyruvate, 2 mm of glutamine, and 10 mM of glucose, and the other filled with 100 µm of carboxy SNARF‐4F dye in the same buffer. Droplets traveled through a PTFE tubing and were collected in a microtube. The droplet mixture was well mixed while maintaining their stability and injected into a PDMS observation chamber with 40 µm height and 1 × 1 cm^2^ dimension by using a pipette. Mixed droplets arranged in a single layer for ease of observation were monitored for 3 h under the bright field microscopy and through 600, and 667 nm wavelength fluorescence microscopy.

### Cell Culture

EL4 (TIB‐39) mouse lymphoma cell line was purchased from American Type Cell Culture (VA, USA). The thawed cell pellet was suspended in RPMI 1640 supplemented with 10% v/v fetal bovine serum and 1%(v/v) penicillin‐streptomycin. The cell suspension was seeded in a Corning T‐75 flask at density of 1.5 × 10^5^ cells mL^−1^ with the total volume of 30 mL and cultured at 37 °C and 5% CO_2_ in a humidified incubator, with the medium changed every 2–3 days. The cell suspension was collected by centrifugation at 200 rcf for 10 min and the supernatant was removed. The cell pellet was resuspended in a live cell imaging solution or Seahorse XF RPMI assay medium to a density of 2.4 × 10^7^ cells/mL for cell encapsulation experiments.

### Cell Encapsulation and Trapping

Cell suspension with a density of 2.4 × 10^7^ cells mL^−1^ was prepared into two, and the two groups of cells were stained with Calcein AM and CellTrace Violet, respectively. The number of cells was dictated to achieve ≈30% of droplet containing single cell generation following a Poisson distribution shown in Equation (1).

(1) $$P\;\left(X\right)=\frac{\lambda^{X}e^{-\lambda }}{X!}\;$$

P is the probability for the number of cells in a droplet. X is the number of cells in a droplet. λ is the mean number of cells per droplet and is calculated by multiplying cell concentration by the droplet volume.^[^ ^46^ ^]^

For the encapsulated cells, glucose supplemented XF medium was used to detect extracellular pH changes during imaging. Cell encapsulation was performed twice to generate two different droplets: one containing 10 µm of oligomycin for hyperglycolytic cells, and the other without oligomycin serving as a control (untreated cells). To prepare hyperglycolytic cell model, Calcein AM‐stained cells were encapsulated by using two aqueous solutions, one filled with 100 µM of carboxy SNARF‐4F dye and 20 µM of oligomycin, and the other one filled with 2.4 × 10^7^ cells mL^−1^. For untreated cells, an equal amount of dimethyl sulfoxide (DMSO), which is a solvent for oligomycin stock solution, was added to the carboxy SNARF‐4F‐containing solution. According to the method 2.3., uniform droplets were generated and collected in a microtube. The droplet mixture was well mixed and injected into a microwell array by using a syringe and a syringe pump to maintain the droplets stably with a slow flow rate. The subsequent process was carried out according to the method 2.3.

### Time‐Lapse Microscopy Imaging

Imaging started immediately after the droplets were captured in the microwell array and was performed using a Zeiss Observer 7 inverted microscope with a motorized stage. The microwell array was located in an environmental chamber that maintained cell culture environment with 95% humidity, 37 °C, and 0% of CO_2_ (Okolab, Pozzuoli, Italy). Excitation light was provided by a LED source (Colibri 5, Carl Zeiss, Germany). Fluorescence for the specific channels was recorded using a multifilter (385, 469, 555, and 631 nm) appropriate band‐pass filters (carboxy SNARF‐4F‐Cy3: Emission at 600 nm, bandwidth 32 nm; carboxy SNARF‐4F‐Cy5: Emission at 667 nm, bandwidth 30 nm; Chroma Technology Corp. USA) and camera settings (Andor ZL41 Cell 4.2 sCMOS, Oxford Instruments, UK) at room temperature (25 °C) and ambient oxygen concentration with 0% of CO_2_. Images were acquired using a 10X objective (Plan‐Apochromat, NA 0.45, Zeiss, Germany). To verify the technology, three positions out of 240 positions of an array were acquired every 5 min in three channels over 3 h. One position in the array was imaged in three channels, and the position was changed afterward and imaged repeatedly. For the 240 position imaging, we used a custom journal of MetaMorph software to obtain images from an entire device automatically by imaging loops. 240 positions in the microwell array were imaged in three channels in a loop of imaging, and imaging was repeated for 3 h. The temporal resolution of the imaging is 14 min.

### Image Analysis

Prior to image analysis, all images were sorted by a custom Python script based on the number of positions. The images were then analyzed using a Fiji free software with Mosaic Particle Tracker plugin and a custom MATLAB script (Mathworks). The details of the parameters used in the Mosaic Particle Tracker were: radius = 10, cutoff = 0, per/abs = 0.6, link = 4, displacement = 5, and dynamics = “Brownian”. Encapsulated cells in the droplets were detected in Green and UV images using a custom Fiji script for automated and quantitative single‐ and multiple cells (*n* > 1) tracking.^[^ ^47^ ^]^ For each position, the custom MATLAB script then generated a binarized droplet mask for 37 images over 3 h and mapped each cell from the Particle Tracker into the droplets based on the tracked cell x and y coordinate position. The script additionally extracted the fluorescence intensities of the tracked cells in single cell droplets in Green/UV, carboxy SNARF‐4F‐Cy3, and carboxy SNARF‐4F‐Cy5 channels. The Green or UV signals were normalized to remove the bleaching of dyes. Fluorescent intensities of empty droplets were sorted out separately. The mean fluorescence intensities of droplets collected from carboxy SNARF‐4F‐Cy3 and carboxy SNARF‐4F Cy5 images were used to calculate a pH value following the manufacturer's protocol.^[^ ^48^ ^]^

### Data Availability

The scripts used in the paper are freely available on GitHub for file organization (https://github.com/kimpenn/microscope‐utility) and image analysis (https://github.com/HyejoongJeong/UPenn_pH).

### Statistical Analysis

At least three independent data points were used for the statistical analysis. The data in the graphs represent mean values, with error bars indicating standard deviations. Statistical significance between test groups was assessed using a two‐tailed Student's *t*‐test. Symbols denote levels of statistical significance (ns: *p* > 0.05, ^*^: *p* ≤ 0.05, ^**^: *p* ≤ 0.01, and ^***^: *p* ≤ 0.001). Outliers were manually evaluated based on the droplet images at position 1 and 100. The criteria for excluding errors from the dataset were established through manual analysis. Errors were then automatically excluded based on the ΔpH criteria (For single cell droplet data, single cell droplet: 0.28 ≤ ΔpH ≤ 1.02; empty droplet: ΔpH < 0.28; multiple cell droplet: ΔpH > 1.02, For empty droplet data, empty droplet: ΔpH ≤ 0.37; error: ΔpH > 0.37). Statistical analysis was performed using Excel for Microsoft 365 (version 16.0).

## Conflict of Interest

The authors declare no conflict of interest.

## Author Contributions

H.J., S.H.H., D.L., and J.K. designed the study. H.J. implemented experiments, analyzed the data, and wrote the manuscript. E.A.L.P. and B.L. created the image analysis pipeline. D.K. wrote the image file sorting script. The other authors assisted with the experiments and discussed the results. All authors have read and approved the final version of the manuscript.

## Supporting information



Supporting Information

Supplemental Video 1

## Data Availability

The data that supports the findings of this study are available from the corresponding authors upon reasonable request.
