# Supplementary material for: SCO‐ph: Microfluidic Dynamic Phenotyping Platform for High‐Throughput Screening of Single Cell Acidification
Source: Small. 2025 Jun 16;21(32):2504687. doi: 10.1002/smll.202504687 (PMC12366287; doi:10.1002/smll.202504687)
Supplement: Supplementary file 1 — Supporting Information [file SMLL-21-2504687-s001.docx]

Supporting Information

**SCO-pH: Microfluidic Dynamic Phenotyping Platform for High-Throughput Screening of Single Cell Acidification**

*Hyejoong Jeong^1,2^, Emilia A. Leyes Porello^1^, Jean G. Rosario^2^, Da Kuang^3^, Syung Hun Han^4^, Jai-Yoon Sul^5^, Bomyi Lim^1^, Daeyeon Lee^1,^*, and Junhyong Kim^2,^**

List of Figures

Materials and methods

Figure S1. Microfluidics devices information for single cell encapsulation.

Figure S2. Droplet uniformity analysis

Figure S3. Microfluidics devices information for microwell array and imaging

Figure S4. Absorption and emission spectra of carboxy SNARF-4F

Figure S5. Optical information of carboxy SNARF-4F (a fluorescent pH probe), laser, and filters.

Figure S6. Microscope organization for droplet-based extracellular pH sensing technology

Figure S7. pH-reducing effect of CO_2_ on live cell imaging solution

Table S1. Comparison of buffer compositions

Figure S8. Buffer selection for long-term pH monitoring

Video S1. Cell tracking in droplets by particle tracking analysis program.

Figure S9. HG and UT droplet discrimination method details.

Figure S10. Calibration curve of 50 μM of carboxy SNARF-4F in supplemented XF solution

**
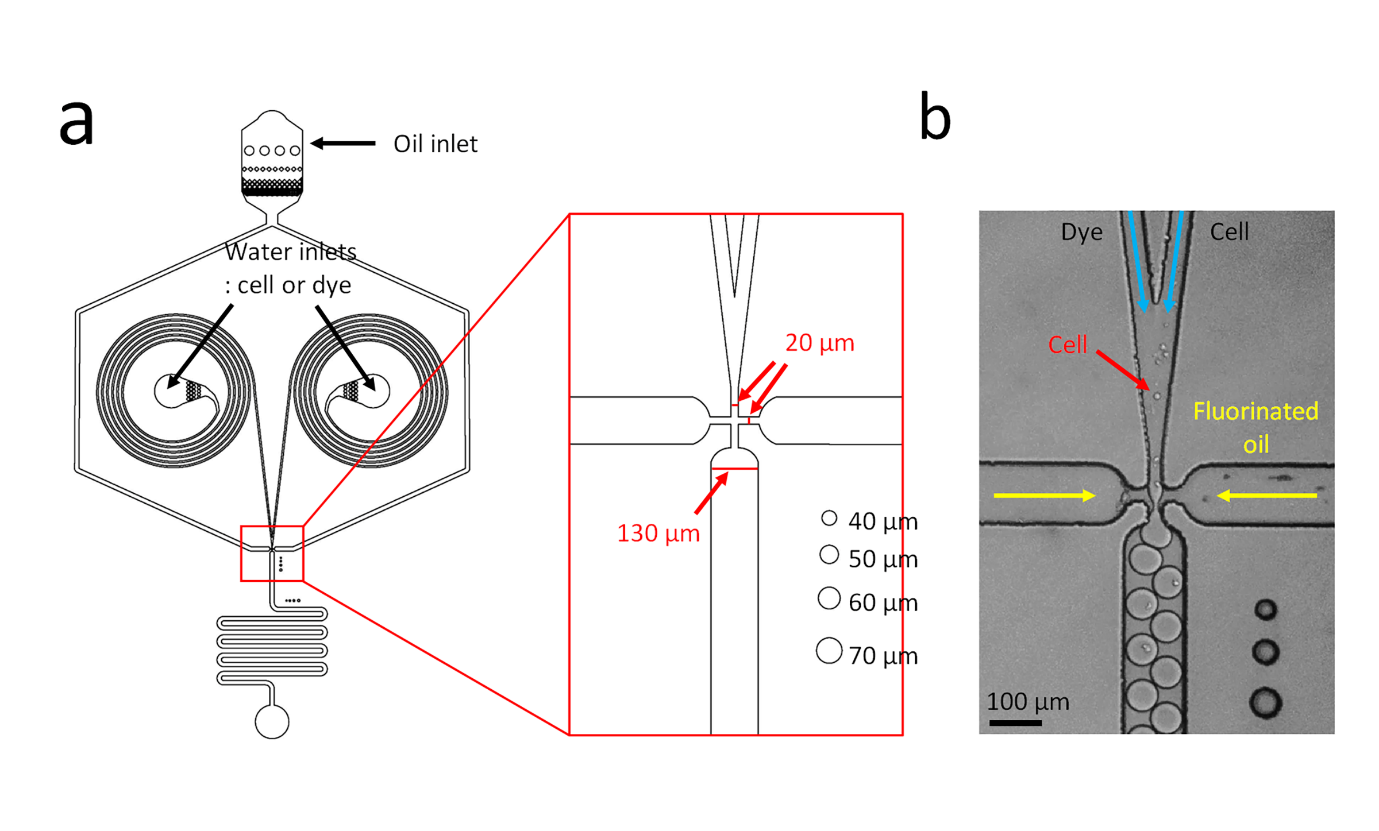
**

**Figure S1.** Microfluidics devices information for single cell encapsulation.

(a) Droplet generator photomask design generated by AutoCAD program. The red box shows an enlarged T-junction, and the detailed channel size and scale bars for droplets are indicated in the box. (b) The image of polydimethylsiloxane (PDMS)-based droplet generator captured by a high-speed camera while droplet generation. The direction of water and fluorinated oil solutions are indicated in the image. The generator has two water inlets, one for dye and one for cells. Fluorinated oil is injected into the oil inlet with some filter features on the top of the generator. Oil comes to the junction horizontally, cutting the aqueous solution to generate droplets. The droplet generator height is 40 μm.


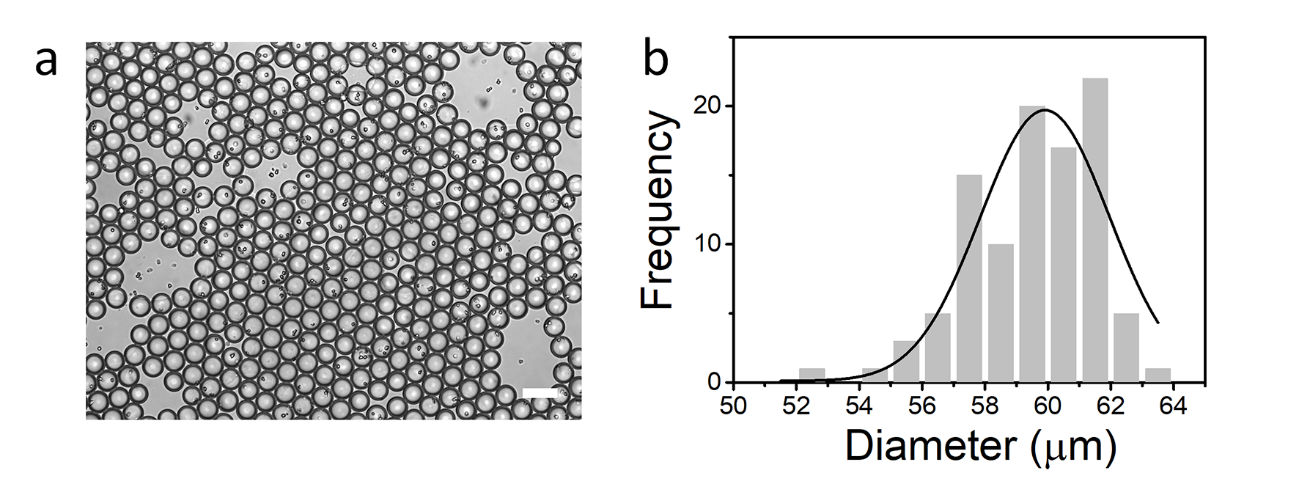


**Figure S2.** Droplet uniformity analysis

1. Droplet distribution image (Scale bar: 100 μm), (b) Droplet size distribution.


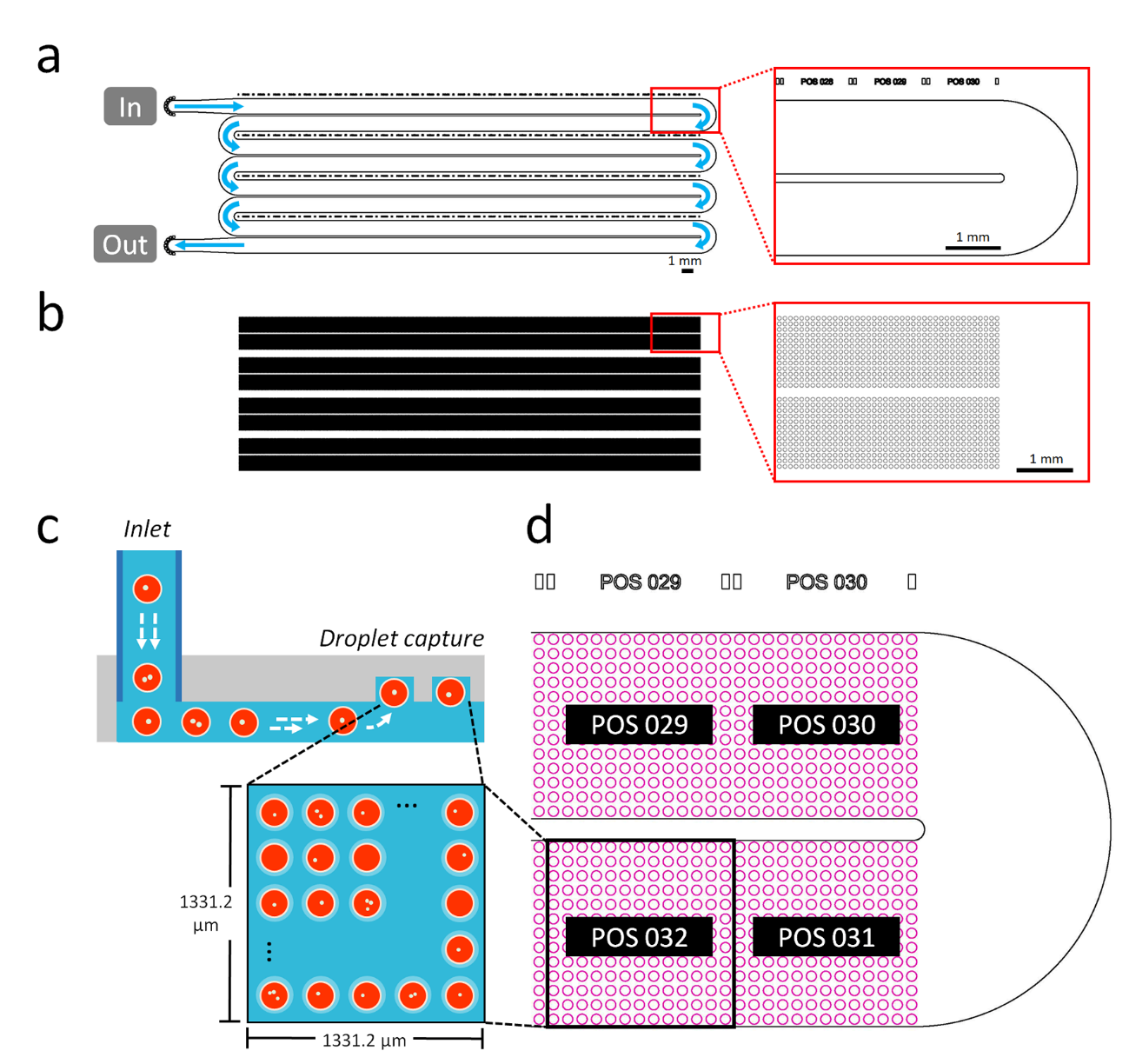


**Figure S3.** Microfluidics devices information for microwell array and imaging

Microwell array photomask design with two layers: (a) a bottom droplet flowing channel and (b) a top microwell array. The height of the droplet flowing channel is 60 μm. Inlet and outlet are displayed in the left image, and the light blue arrows indicate direction of flow. A small red box is enlarged on the right side to present two lane pair and position numbering above the first lane. (b) The height of a microwell is 50 μm (110 μm from the bottom). A small red box is enlarged on the right side to present two lane pair, and the lane containing small circles, which are microwells. (c) Schematic illustration of the cross-section of the microwell array. Droplets enter the inlet and are automatically captured into microwells above the flowing channel due to the density difference between water and fluorinated oil. The imaging window indicated by the black square contains 169 microwells (13 x 13) and is viewed from the top of the microwell. This imaging window corresponds to (d) “Position 32” on the photomask design. A small microwell array includes 240 positions for imaging.

**
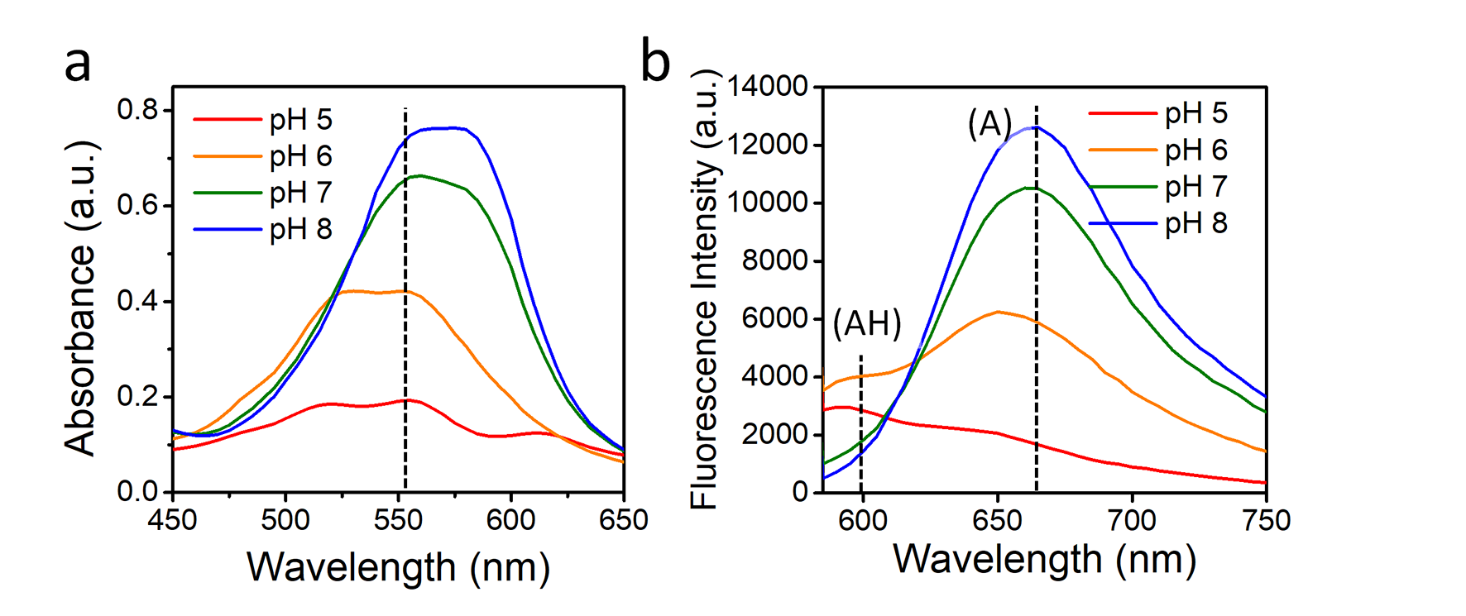
**

**Figure S4.** Absorption and emission spectra of carboxy SNARF-4F dissolved in 50 μM in phosphate buffered saline at various pH values by using a plate reader.

(a) Absorption spectra indicating maximum absorbance at 550 nm. (b) Fluorescence intensities obtained by excitation at 555 nm. The ‘AH (acid)’ form exhibits an emission peak at 600 nm, while the ‘A (base)’ form emits at 667 nm.


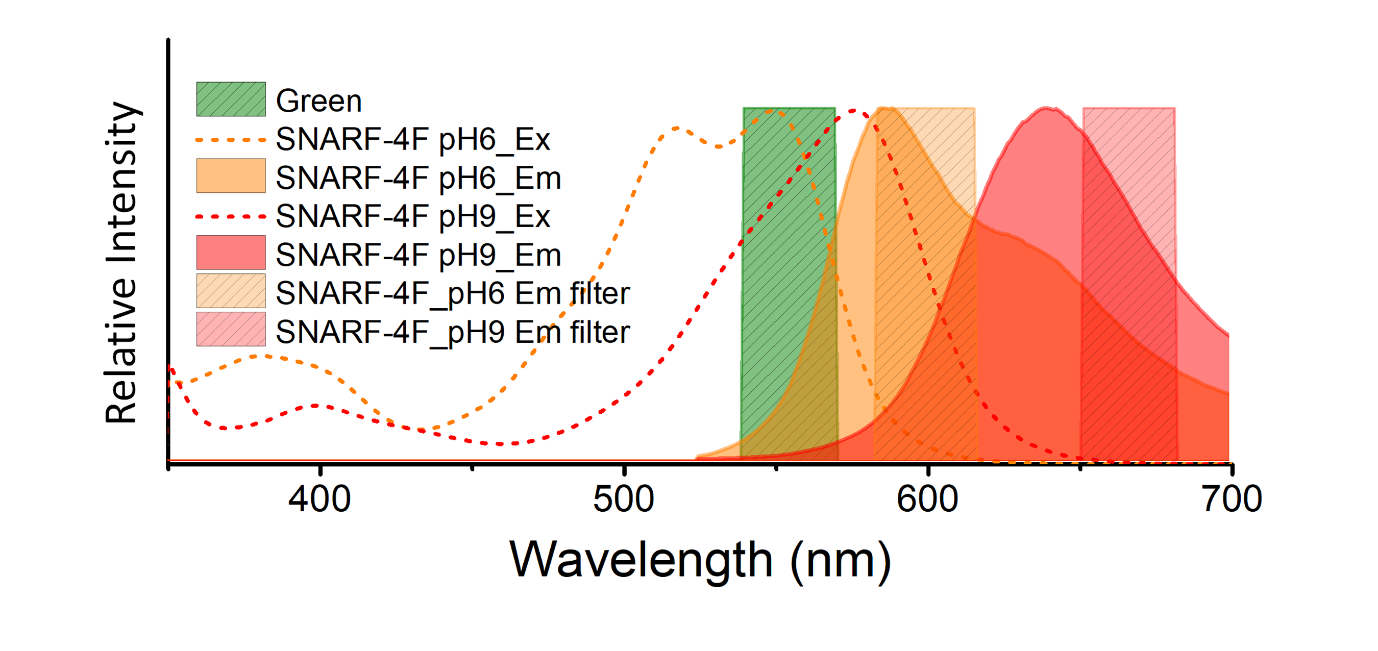


**Figure S5.** Optical information of carboxy SNARF-4F (a fluorescent pH probe), laser, and filters.

The green hatched graph indicates a green laser. The dotted lines are excitation plots of carboxy SNARF-4F at pH 6 (orange) and pH 9 (red). The solid lines with color-filled area are emission plots of carboxy SNARF-4F at pH 6 (orange) and pH 9 (red). The hatched graphs indicate emission filters for carboxy SNARF-4F at pH 6 (orange) and pH 9 (red). Ex, excitation; Em, emission.


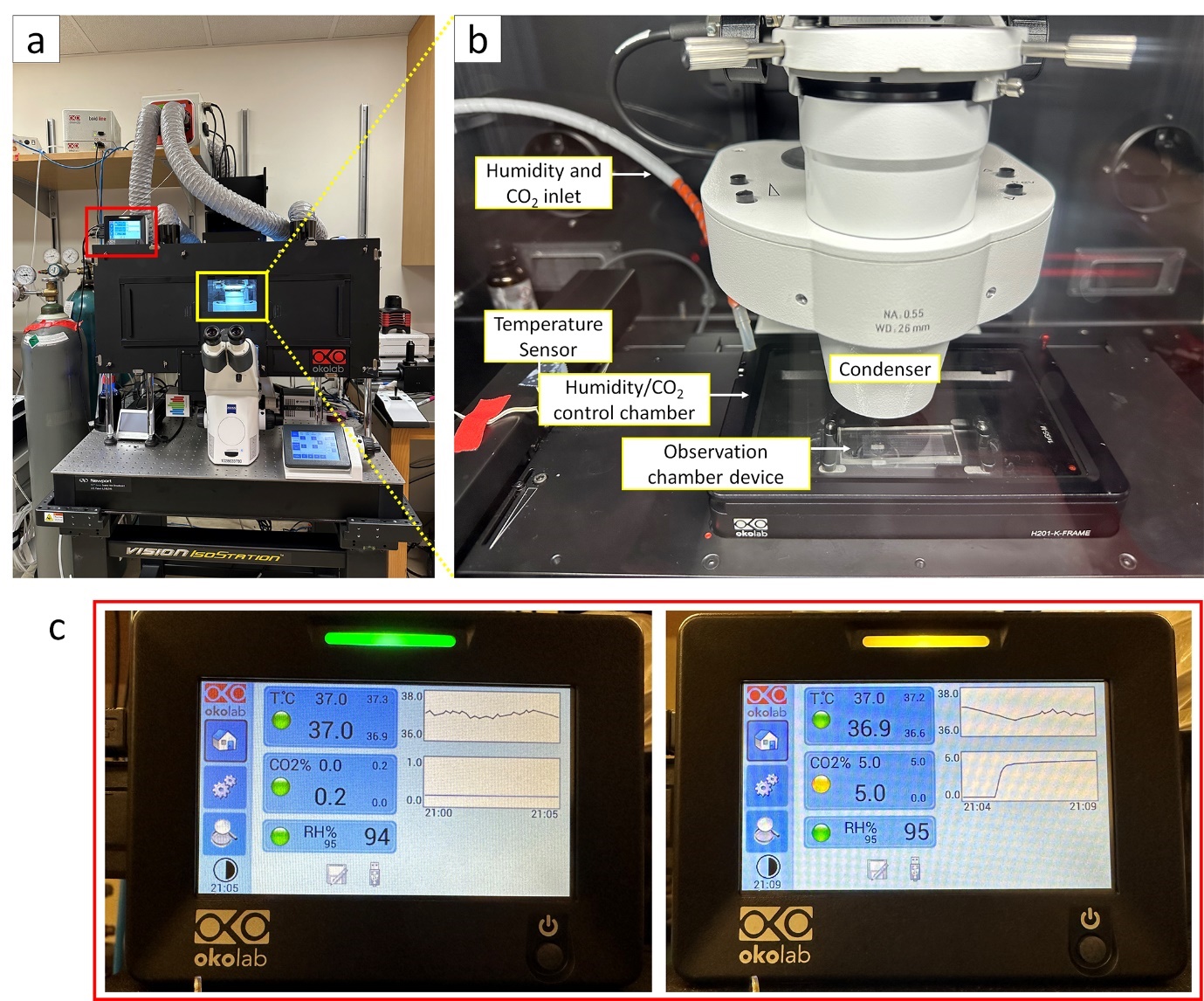


**Figure S6.** Microscope organization for single cell optical pH analysis technology

(a) The entire inverted microscope body is enclosed within an environmental chamber capable of controlling temperature, humidity, and CO_2_ concentration. (b) Inside view of the environmental chamber through the front window. The chamber maintains a temperature of 37°C and is kept dark. The temperature is monitored by a temperature sensor within the environmental chamber, while humidity and CO_2_ concentration are controlled in a separate smaller chamber located on the stage. Inlets for humidity and CO_2_ are connected to this chamber. The PDMS-based microwell array is positioned inside the humidity/CO_2_ chamber and secured to the stage using magnets. (c) The observation environment is displayed in real-time on the monitor of the environmental chamber. Changes in CO_2_ concentration from 0% to 5% can be rapidly applied to this equipment.


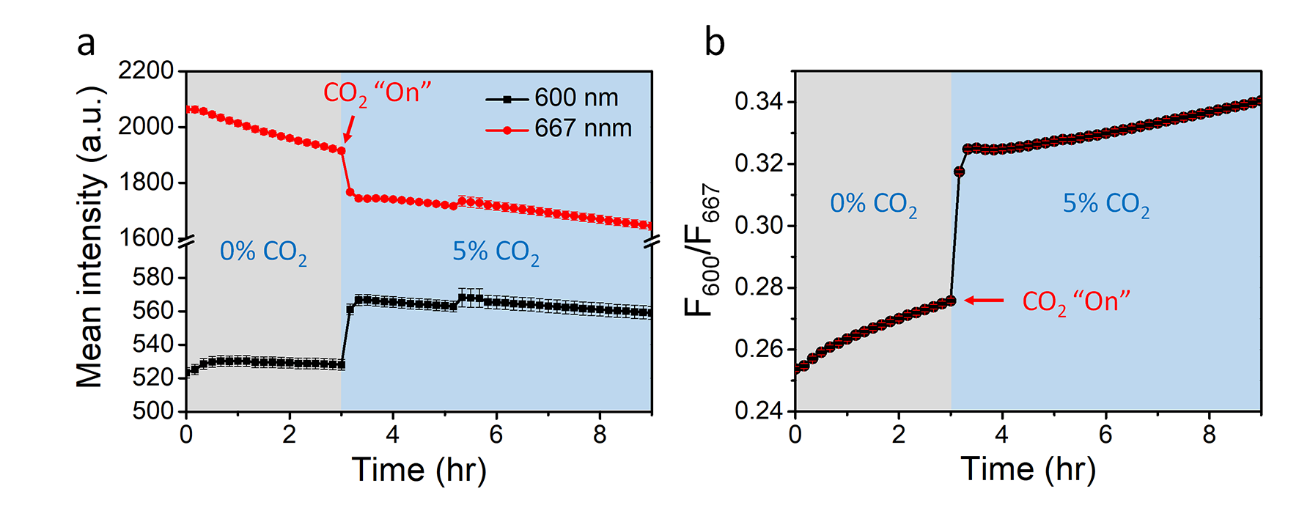


**Figure S7.** pH-reducing effect of CO_2_ on live cell imaging solution (LCIS).

Droplets containing 50 μM carboxy SNARF-4F in LCIS were generated and loaded into the microwell array. The array was observed at 37°C, 95% humidity, and 0% CO_2_ for 3 hours, and then subsequently observed at 5% of CO_2_ for another 6 hours. One position out of 240 positions in the chamber was measured at 10-min intervals for a total of 9 hours. Intensities at 600 nm and 667 nm of each droplet were collected using a custom MATLAB code. (a) Mean intensity of 169 droplets at one position at 600 nm and 667 nm with standard error of the mean. (b) Mean intensity ratio between 600 nm and 667 nm for each droplet with standard error of the mean. Table S1. Comparison of buffer compositions: PBS, live cell imaging solution (LCIS), and Seahorse XF RPMI medium.

| Concentration (mM) | PBS 1X (pH 7.4) | LCIS (pH 7.4) | Seahorse XF RPMI medium pH 7.4 |
| --- | --- | --- | --- |
| NaCl | 58 | 140 | 136.9 |
| KCl | - | 2.5 | 5.4 |
| HEPES | - | 20 | 1.0 |
| KH_2_PO_4_ | 136 | - | - |
| Na_2_HPO_4_-7H_2_O | 268 | - | - |
| NaHPO_4_ | - | - | 5.6 |
| CaCl_2_ | - | 1.8 | - |
| MgCl_2_ | - | 1.0 | - |
| Ca(NO_3_)_2_ | - | - | 0.4 |
| MgSO_4_ | - | - | 0.4 |


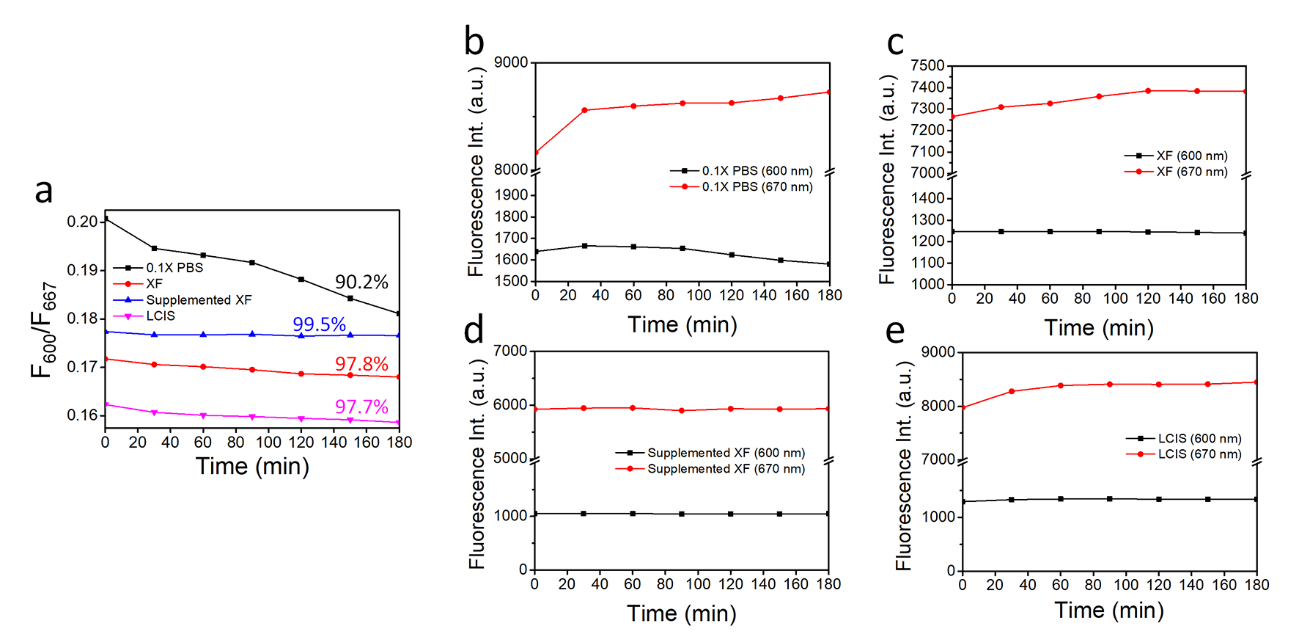


**Figure S8.** Buffer selection for long-term pH monitoring (37°C, 0% humidity, 0% CO_2_)

To accurately measure extracellular pH in tiny droplets, buffer stability under a long-term observation condition has been assessed using 0.1X PBS, Seahorse XF RPMI medium (XF), supplemented XF RPMI medium, and live cell imaging solution (LCIS). XF RPMI medium is supplemented by 2 mM of GlutaMax, 10 mM of glucose, and 1 mM of sodium pyruvate according to the reference.[1]

50 μM of carboxy SNARF-4F was prepared in four different buffer conditions and was loaded into the Secure-Seal^TM^ hybridization chamber for observation under the microscope. Imaging was performed at 600 nm and 667 nm for 3 hours at 5 min intervals. After imaging, fluorescence intensities at each wavelength were compared. (a) The ratio of fluorescence intensities at 600 nm (F_600_) and 667 nm (F_667_) of four different buffer conditions. Fluorescence intensities at 600 nm and 667 nm of (b) 0.1X PBS, (c) XF, (d) supplemented XF medium, and (e) LCIS.

**Video S1. Cell tracking in droplets by particle tracking analysis program.**

Hyperglycolytic (HG) cells are detected by the GFP channel, and untreated (UT; normal glycolysis) cells are detected by the DAPI channel. Position 1 (a yellow box in Figure 4) shows three droplets containing HG multiple cells, HG single cell, and UT single cell in order from left to right. Position 2 (a red box in Figure 4) shows three droplets containing UT multiple cells, UT single cell, and no cell in order from left to right.


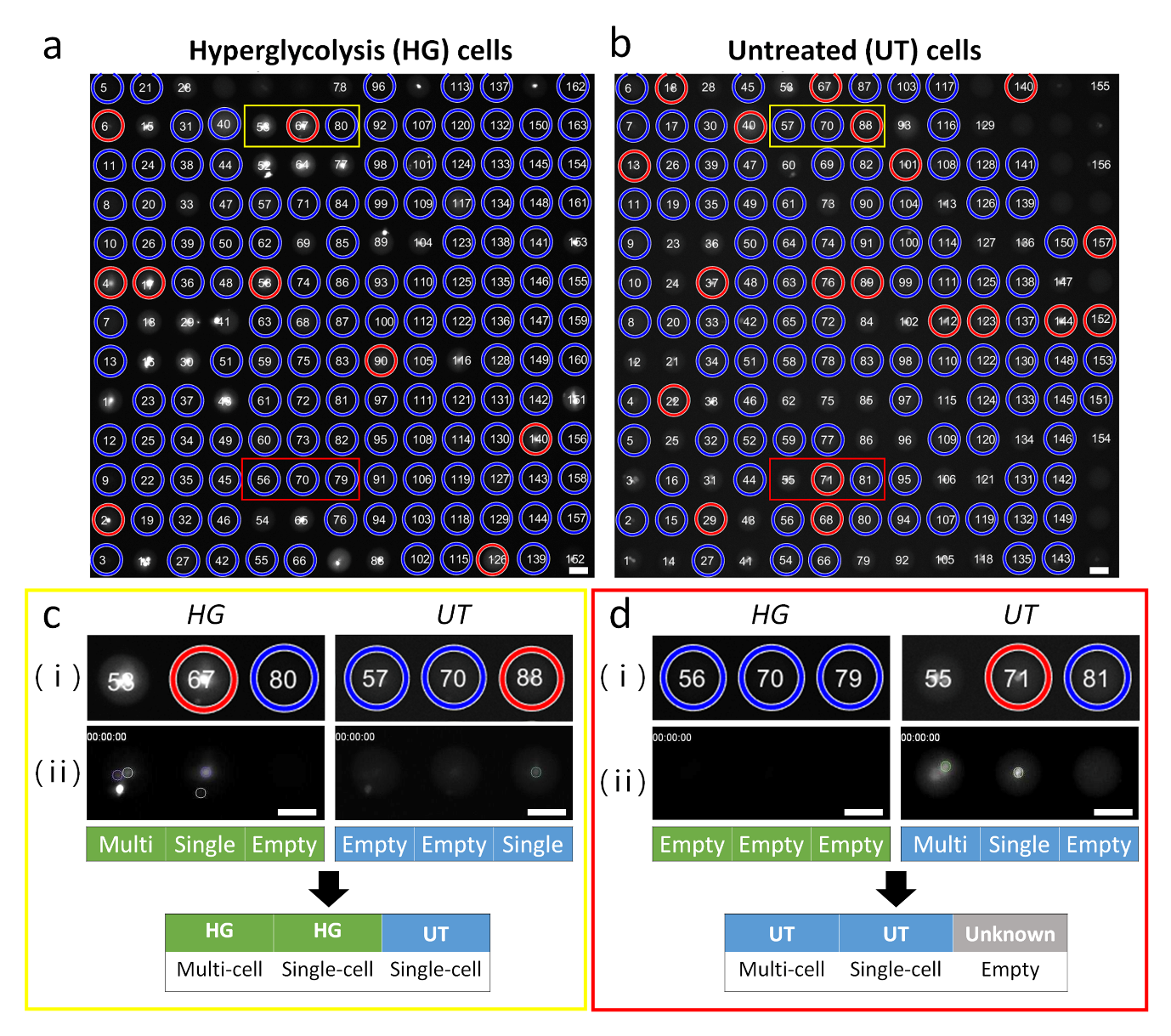


**Figure S9.** HG and UT droplet discrimination method details. Droplet indexing results are obtained from MATLAB: (a) Hyperglycolytic (HG) cells are detected at the GFP channel, and (b) untreated (UT) cells are detected by the DAPI channel. Droplets containing a single cell are marked with a red circle, empty droplets are marked with a blue circle, and droplets containing multiple cells are unmarked. For examples of pH monitoring performance, three droplets in the yellow (Position 1) and red (Position 2) boxes in both images are magnified in (c) and (d), respectively. (c) (ⅰ) Magnified droplet indexing images of HG cells and UT cells, along with (ⅱ) the images of detected cells by the Particle Tracker are displayed. Detected single cells are outlined in the representative image at the beginning of imaging (0h). The type of droplet (Hyperglycolytic cells vs. Untreated cells; HG vs. UT) and number of cells (Single vs. Multi vs. Empty) are indicated in the table. (d) (ⅰ) Magnified droplet indexing images of HG cells and UT cells in the red box. (ⅱ) Images of the detected cells by the Particle Tracker are displayed below the droplet indexing images. Cells are outlined in the image at the beginning of imaging (0h). The scale bar is 50 μm.

To enhance the reader’s understanding, we show how we distinguish droplets containing HG cells and UT cells that are randomly immobilized in a microwell array. By comparing droplet indexing results of GFP and DAPI images, which are corresponding to HG cells and UT cells. We can know that three droplets in a yellow box are not HG multi cell droplet, HG single cell droplet, and empty droplet and are HG multi cell droplet, HG single cell droplet, and UT single cell droplet. In the red box, only UT cells are observed. Three droplets are UT multi cell droplet, UT single cell droplet, and empty droplet. Based on these results, we determine that this technique can monitor single cell acidification with diverse glycolytic metabolism.


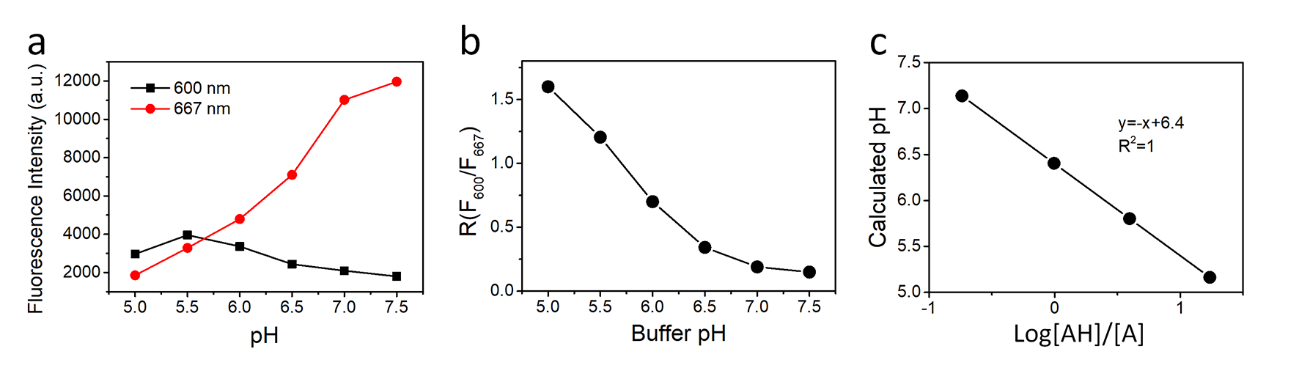


**Figure S10.** The calibration curve of 50 μM of carboxy SNARF-4F in XF unbuffered solution with supplements obtained by a fluorescence microscope.

(a) Fluorescence intensities at 600 nm and 667 nm at pH ranging from 5 to 7.5. The excitation laser was 555 nm. (b) The ratio of fluorescence intensities at 600 nm and 667 nm (R(F_600_/F_667_)) at various buffer pH. (c) The plot of the calculated pH and the decimal logarithm of the reciprocal of the hydrogen ion activity.

(1)

$\left[ H^{+} \right]=K_{a}\left( \frac{R-R_{B}}{R_{A}-R} \right)\times\frac{F_{B(\lambda2)}}{F_{A(\lambda2)}}$ (1)

$pH=pK_{A}-\log\left[ \frac{R-R_{B}}{R_{A}-R}\times\frac{F_{B(\lambda2)}}{F_{A(\lambda2)}} \right]$ (2)

The pH-dependent shift of carboxy SNARF-4F allows calibration of the pH response using the dual-emission ratio with 𝜆_1_=600 𝑛𝑚 and 𝜆_2_=667 𝑛𝑚 (**Figure S9a**). R is the ratio 𝐹_𝜆1_/𝐹_𝜆2_ of fluorescence intensities (*F*) measured at two different wavelengths 𝜆_1_ and 𝜆_2_ **(Figure S9b)**. The subscripts A and B represent the limiting values at the acidic and basic endpoints of the titration, respectively. 𝐹_𝐵(𝜆2)_/𝐹_𝐴(𝜆2)_ represents the normalization factor. Equation (2), the logarithmic form of equation (1), yields a linear plot with a slope of 1 and an intercept equal to the pKa 6.4 (shown in **Figure S9c**). From this result, we determined that the ratio (F_600_/F_667_) is a linear function of pH of the solution between pH 5.0-6.5 and accurately reflects pH change. The calculated pH exhibited minimal deviation from the buffer pH, with discrepancies of 6.1%, 3.3%, 1.5%, and 2.0% at pH values of 5.5, 6.0, 6.5, and 7.0, respectively. Therefore, we conclude that carboxy SNARF-4F provides accurate pH detection from droplets. All droplet pH values were calculated using equation (2).

Reference

[1] Rivadeneira, D.B., et al., Survivin promotes oxidative phosphorylation, subcellular mitochondrial repositioning, and tumor cell invasion. Science signaling, 2015. 8(389): p. ra80-ra80.
